# In vitro evaluation of enamel bond strength of an orthodontic adhesive enhanced with 2 wt% nano-hydroxyapatite derived from Asian Moon Scallop (*Amusium pleuronectes*)

**DOI:** 10.2340/biid.v12.45130

**Published:** 2025-12-17

**Authors:** Gufa Bagus Pamungkas, Eddy Heriyanto Habar, Maria Tanumihardja

**Affiliations:** a Doctoral Program, Faculty of Dentistry, Hasanuddin University, Makassar, Indonesia; b Department of Orthodontics, Faculty of Dentistry, Hasanuddin University, Makassar, Indonesia; c Department of Conservative Dentistry, Faculty of Dentistry, Hasanuddin University, Makassar, Indonesia

**Keywords:** Orthodontic adhesive, nano-hydroxyapatite, Asian moon scallop, shear bond strength, adhesive remnant index

## Abstract

**Objective:**

Bracket detachment remains a frequent complication in orthodontic treatment, often resulting in extended treatment duration and increased clinical workload. Nano-hydroxyapatite (n-HAp), due to the excellent biocompatibility and bioactivity, has been explored as a potential filler to improve the performance of dental adhesive systems. This study aimed to evaluate the effect of incorporating 2 wt% n-HAp derived from *Amusium pleuronectes* (Asian moon scallop) shells into orthodontic adhesive on shear bond strength (SBS) to enamel and on Adhesive Remnant Index (ARI) scores.

**Materials and Methods:**

Thirty-two extracted human maxillary premolars were randomly divided into two groups (*n* = 16). The experimental group used orthodontic adhesive modified with 2 wt% n-HAp, while the control group used unmodified commercial adhesive. Standard bracket bonding procedures were performed, followed by SBS testing using a universal testing machine. After debonding, the adhesive remnants were evaluated under a stereomicroscope to determine ARI scores. SBS data were analyzed using an independent t-test, and ARI scores were assessed using the Mann–Whitney U test, with significance set at *p* < 0.05.

**Results:**

The experimental group exhibited a significantly higher mean SBS of 16.54 ± 2.98 MPa compared to 8.91 ± 1.63 MPa in the control group (*p* < 0.05). ARI scores showed no statistically significant difference between the two groups (*p* > 0.05), with mean scores of 3.44 ± 1.23 and 3.38 ± 1.22, respectively.

**Conclusion:**

Incorporation of 2 wt% nano-hydroxyapatite derived from Asian moon scallops into an orthodontic adhesive improved bond strength without altering failure patterns, suggesting its potential as an effective bioactive filler in orthodontic applications.

## Introduction

Fixed orthodontic appliances remain a cornerstone in the management of malocclusions and complex dental discrepancies [1]. Despite advances in bracket and adhesive technology, orthodontic bracket detachment remains a prevalent clinical problem, with an incidence rate reported to be as high as 32.1%. This complication not only compromises the efficiency and duration of treatment but also imposes additional clinical chair time and patient inconvenience [2]. The likelihood of bracket detachment tends to increase proportionally with the length of treatment, often necessitating multiple rebonding procedures. These events not only interfere with planned tooth movement but may also lead to cumulative enamel damage and increased treatment costs [3].

A critical factor influencing bracket retention is the bond strength of the orthodontic adhesive system [3–5]. Previous research determined that the minimum for orthodontic shear bond strength (SBS) is 6–8 MPa to withstand masticatory and orthodontic forces, and higher bond strengths mean that greater force can be withstood [6]. On the other hand, the bond strength of orthodontic adhesives should not be too high to avoid enamel loss during the debonding procedure (40–50 MPa). Therefore, the optimal orthodontic biomaterial should exhibit bond strengths within the range of 6 to 50 MPa, although these values are primarily theoretical [5]. Consequently, achieving an optimal balance between adequate mechanical retention and patient safety remains a priority in adhesive development [7].

Filler content within the orthodontic adhesive is recognized as a key variable affecting the mechanical performance, including strength, viscosity, polymerization shrinkage, and handling characteristics [8]. Specifically, the inclusion of inorganic fillers can significantly enhance the mechanical integrity of the resin matrix by improving stress distribution and reinforcing the bonding interface [9]. Considering that orthodontic adhesives should not have excessive bond strength, the addition of organic fillers must be controlled. Previous research has added nanoparticles as a filler into orthodontic adhesives using the direct mixing method with a ratio of 2% and 4% by weight, comparing with the control group (standard orthodontic adhesive) [10]. The results of the study showed that the addition of 2% by weight of nanoparticles as a filler was recommended, with the result being an increase in bond strength within the safe limits without adversely affecting viscosity or handling characteristics of the orthodontic adhesive, whereas the addition of 4% nanoparticles as a filler decreased bond strength significantly [10].

One promising class of fillers currently being explored is nano-hydroxyapatite (n-HAp) [11]. Owing to the chemical and structural similarity to natural enamel and bone, n-HAp exhibits excellent biocompatibility, bioactivity, and remineralization potential [12]. Incorporation into dental adhesives and restorative materials has shown potential to enhance mechanical strength, bond integrity, and biological interactions at the tooth–material interface. In contrast to microsized hydroxyapatite, the nanoscale dimensions of n-HAp facilitate greater surface area and more intimate contact with surrounding tissues, leading to improved performance in biomaterial applications [13].

Hydroxyapatite can be synthesized from both synthetic and biowaste-derived sources [11]. However, biowaste-derived sources are increasingly favored due to their low cost, ecofriendliness, and higher biological affinity [14]. Marine biowaste, particularly mollusk shells, has emerged as a sustainable and abundant source of calcium carbonate (CaCO₃), the primary precursor in hydroxyapatite synthesis [15]. Among these, the Asian moon scallop (*Amusium pleuronectes*) is widely harvested in Southeast Asia, including Indonesia. While the scallop meat is consumed, the shells are typically discarded as waste, contributing to environmental pollution and resource underutilization [16].

Preliminary studies have demonstrated the feasibility of converting Asian moon scallop shells into n-HAp through environmentally friendly wet chemical precipitation methods. This approach not only adds value to marine biowaste but also aligns with principles of green chemistry and circular economy [17]. The resulting n-HAp has been found to possess the following characteristics: classified under nanoparticle size category (88–433 nm), favorable morphology, high crystallinity, superior biointegration potential, and no agglomeration occurring between particles because of the ultrasonication procedure carried out, making it a viable candidate for incorporation into dental materials.

The aim of this study was to evaluate the effect on the SBS to enamel and adhesive remnant of incorporating 2% weight n-HAp derived from Asian moon scallop (*Amusium pleuronectes*) shells into an orthodontic adhesive.

## Materials and methods

This study was designed as a laboratory-based experimental investigation and was conducted following ethical approval obtained from the Research Ethics Committee of the Faculty of Dentistry-Dental Hospital, Hasanuddin University (Letter No. 007/KEPK FKG-RSGMP UH/EA/I/2025). The sample size was determined using the Federer formula: (n-1)(t-1) ≥ 15, where *n* is the number of samples per group and *t* is the number of groups. Given two experimental groups, the formula yielded a minimum of 16 samples per group, resulting in a total of 32 samples used in this study.

### Synthesis of nano-hydroxyapatite

The n-HAp was synthesized from Asian moon scallop shells, a marine byproduct rich in calcium carbonate (CaCO₃), using a wet chemical precipitation method. The shells were thoroughly cleaned to remove organic residues, dried, and then ground into a fine powder using a planetary ball mill at 300 rpm for 2 h to achieve uniform particle size distribution. The calcium precursor was obtained by calcining the shell powder at high temperature to convert CaCO₃ into calcium oxide (CaO), which was then reacted with a phosphate source under controlled pH and temperature to precipitate hydroxyapatite at the nanoscale [17]. The synthesized n-HAp was filtered, washed, and dried before being characterized using analytical techniques such as X-ray diffraction (XRD) to determine crystallinity, Fourier-transform infrared spectroscopy (FTIR) for functional group analysis, scanning electron microscopy (SEM) for particle morphology, and energy-dispersive X-ray spectroscopy (EDX) for elemental composition [18].

### Preparation of the experimental adhesive

The experimental adhesive was prepared by incorporating 2% by weight of the synthesized n-HAp into a commercial orthodontic adhesive (Transbond™ XT, 3M Unitek, CA, USA), The 2 wt% n-HAp concentration was selected in accordance with previous studies showing the effectiveness in enhancing adhesive performance without negatively impacting the viscosity or workability of the composite resin [10]. The n-HAp powder derived from Asian moon scallop shells was weighed to 2 wt% of the adhesive mass. The powder was gradually added to the Transbond™ XT adhesive under light-protected conditions inside a dark room. Manual mixing was performed using a clean glass rod for 10 min under controlled conditions until a visually homogeneous paste was obtained. However, uniform nanoparticle dispersion cannot be guaranteed with this method. Advanced dispersion methods such as ultrasonication or rheometry were not performed and are acknowledged as a limitation. To maintain reproducibility, mixing was performed under standardized conditions with controlled duration and environmental factors.

### Tooth preparation and bonding procedure

A total of 32 extracted human upper premolars stored in 0.1% thymol solution were selected based on intact buccal enamel and absence of caries or cracks. Each tooth was embedded in a self-cure acrylic resin block (Hilon, London, England) with standardized dimensions of 25 mm × 25 mm × 7 mm, leaving the buccal surface fully exposed and perpendicular to the base for testing. The teeth were then randomly divided into two groups (*n* = 16 each):

- **Control group:** Brackets bonded using unmodified Transbond XT adhesive.
- **Experimental group:** Brackets bonded using an adhesive containing 2% wt n-HAp.

The tooth surfaces were cleaned with nonfluoridated pumice and water, then etched with 37% phosphoric acid gel for 30 s, rinsed thoroughly, and air dried. A bonding agent (Transbond XT Primer) was applied and light cured for 10 s. Subsequently, the orthodontic adhesive (either Transbond XT adhesive for the control group or the experimental adhesive containing 2 wt% nano-hydroxyapatite) was applied to the bracket base. Stainless steel orthodontic brackets (Ortho Technology, FL, USA) were positioned at the center of the buccal surface and firmly pressed into place. Excess adhesive was carefully removed, and each bracket was light cured for 20 s (10 s mesial and 10 s distal) using an LED curing light.

### Shear bond strength testing

**Figure 1:**
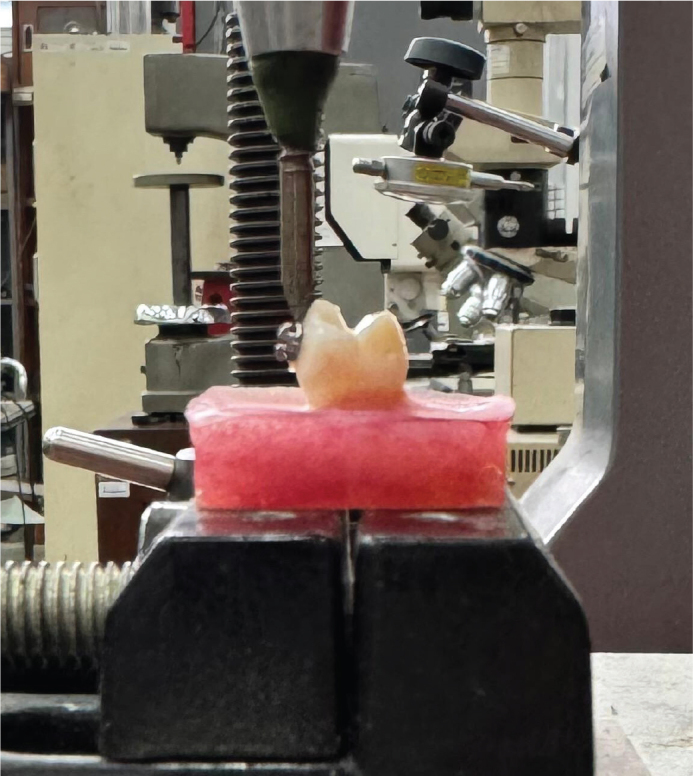
Shear bond strength testing performed using a universal testing machine.

All bonded samples were stored in distilled water at 37°C for 24 h prior to mechanical testing. The SBS test was performed using a Universal Testing Machine (Pearson Panke Equipment, Hertfordshire, England). Each acrylic block was secured in the testing jig so that the blade of the machine applied a force parallel to the bracket–tooth interface and perpendicular to the bracket base at a crosshead speed of 1 mm/min (Figure 1). The load at failure (debonding force) was recorded in Newtons (N). SBS (MPa) was calculated by dividing the force value by the surface area of the bracket base (in mm²) [19].

### Adhesive Remnant Index assessment

**Figure 2:**
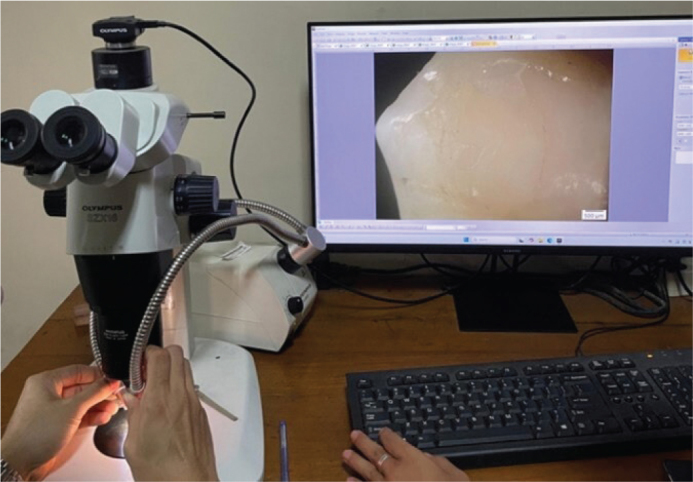
Post-debonding adhesive evaluation under a stereomicroscope.

**Figure 3:**
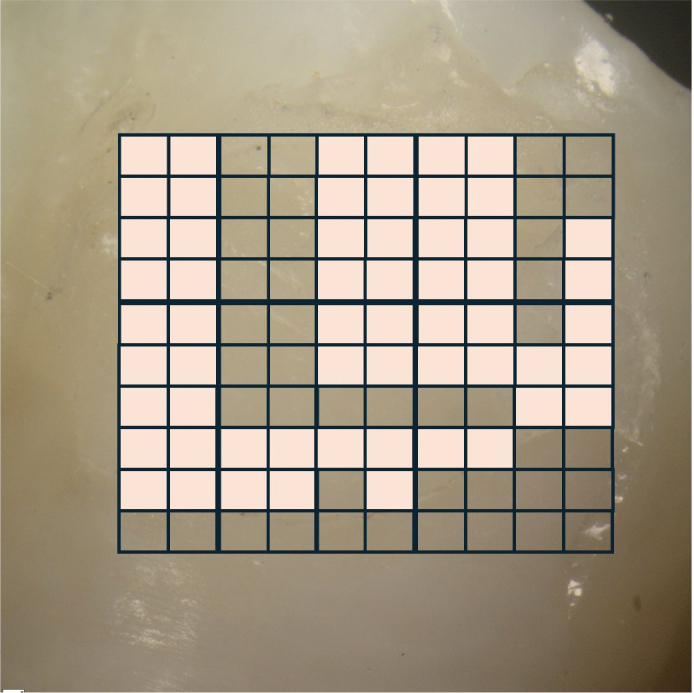
Assessment of adhesive remnant on enamel surfaces (the bold square indicates the area covered with adhesive).

Following debonding, each enamel surface was examined under a stereomicroscope (SZX18, Olympus, Tokyo, Japan) at 10× magnification (Figure 2). ARI scores were recorded by visually dividing the debonded bracket base area into 100 equal parts and estimating the percentage of adhesive material remaining on the enamel surface (Figure 3). The amount of residual adhesive left on the tooth surface was scored using a modified Adhesive Remnant Index (ARI) as follows:

- Score 1: 100% adhesive remaining on the enamel
- Score 2: > 90% adhesive remaining on the enamel
- Score 3: 10–90% adhesive remaining on the enamel
- Score 4: < 10% adhesive remaining on the enamel
- Score 5: No adhesive remaining on enamel [20].

Samples were randomly allocated to groups using a sealed-envelope method to minimize selection bias. All samples were scored twice by the same examiner at two different time points to minimize observational bias. To determine the consistency of the scoring, Cohen’s Kappa statistic was calculated, resulting in a value of κ = 0.844, indicating very good intraexaminer agreement.

### Statistical analysis

All numerical data for SBS were analyzed by an independent t-test to compare the means between groups. The ARI scores, as ordinal data, were compared using the nonparametric Mann–Whitney U test. A p-value less than 0.05 was considered statistically significant for all comparisons.

## Result

**Figure 4:**
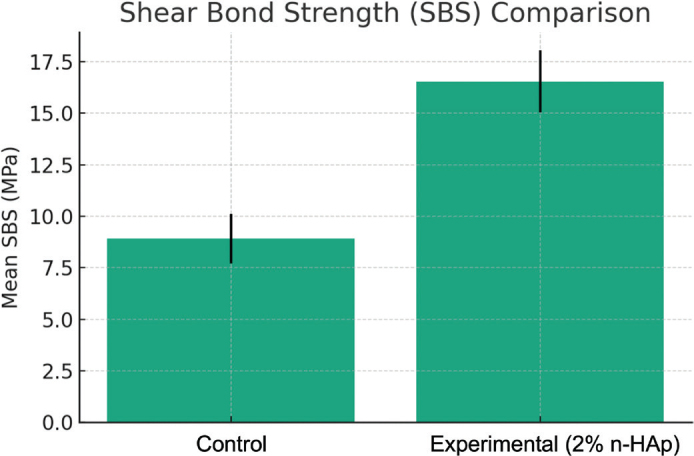
The mean of shear bond strength values.

The SBS results are presented in Figure 4. The experimental adhesive (n-HAp) was found to result in a statistically higher SBS (16.54 ± 2.98 MPa) than the control adhesive (8.91 ± 1.63 MPa) (*p* < 0.05).

The ARI scores are presented in Table 1. The statistical analysis revealed no significant difference between the groups (*p* > 0.05).

**Table 1:** The value of the adhesive remnant index scores.

Group	*n*	Score 1	Score 2	Score 3	Score 4	Score 5	Mean
**Experimental**	16	1 (6.25%)	3 (18.75%)	4 (25%)	4 (25%)	4 (25%)	3.44 ± 1.23
**Control**	16	1 (6.25%)	4 (25%)	2 (12.5%)	6 (37.5%)	3 (18.75%)	3,38 ± 1.22

## Discussion

This study demonstrated a statistically significant increase in SBS of orthodontic brackets following incorporation of 2 wt% n-HAp derived from *Amusium pleuronectes* into an orthodontic adhesive. The mean SBS in the experimental group increased by approximately 85% compared to the control group, aligning with prior reports where n-HAp improved composite resin mechanical properties [11, 21, 22]. These results suggest that n-HAp has the potential to reinforce orthodontic adhesives, thereby enhancing bracket retention.

n-HAp’s nanoscale dimensions and high surface area theoretically facilitate better interfacial interactions with the adhesive matrix. Literature indicates that hydroxyl groups on n-HAp can form hydrogen bonds or other secondary interactions with methacrylate-based monomers, possibly improving polymer network crosslinking and mechanical integrity [17, 23, 24]. Nonetheless, the present study did not employ chemical analyses such as FTIR or Raman spectroscopy to directly confirm such bonding; therefore, these mechanisms remain hypothetical and warrant rigorous experimental verification.

Further, the potential effects of n-HAp on resin rheology and polymerization shrinkage, factors critical to adhesive performance and marginal integrity, were not assessed. Nanofillers may influence viscosity and flow, impacting adaptation to the enamel and bracket base surfaces [25]. However, manual hand-mixing without rheological characterization introduces uncertainty regarding filler dispersion and homogeneity, which could have contributed to variability in SBS values. Advanced microscopy and rheometry should be employed in future work to characterize filler distribution and adhesive flow properties comprehensively.

The 2 wt% filler concentration was selected based on previous research, where 2 wt% n-HAp added to an orthodontic adhesive improved both the degree of conversion and the SBS, whereas 4 wt% showed a decline in performance relative to 2 wt% and sometimes even relative to the control [10]. Other studies have shown that filler content exceeding approximately 5 wt% can significantly increase viscosity, potentially impairing adhesive handling, penetration, and bond strength due to agglomeration or reduced flowability [26]. Conversely, filler concentrations below a certain threshold may fail to confer meaningful mechanical reinforcement. Taken together, these findings highlight the need for a systematic evaluation across a broader range of filler concentrations to identify the optimal balance between mechanical enhancement and clinical usability. Such studies should include viscosity profiling, rheological behavior, filler dispersion analysis, and long-term performance testing to ensure the functional and practical viability of the adhesive formulation.

The ARI scores showed no significant difference between the two groups, with both groups predominantly scoring 4, indicating that less than 10% of adhesive remained on the enamel surface after debonding. This suggests that bond failure mainly occurred at the enamel–adhesive interface, which is clinically advantageous as it minimizes the amount of adhesive left on the tooth. Consequently, this reduces the need for mechanical removal of adhesive with burs or scalers, thereby lowering the risk of iatrogenic enamel damage during cleanup [19]. While ARI scores indicate the site of bond failure rather than bond strength, the higher SBS observed in the experimental group alongside similar failure modes suggests that incorporating 2 wt% n-HAp improved adhesive performance without compromising the safe failure pattern.

This study has several limitations. The relatively small sample size, although determined using Federer’s formula, remains at the lower end for SBS testing. No power analysis and effect size calculation were performed, which may limit the strength and generalizability of the findings. The manual hand-mixing procedure employed for n-HAp incorporation introduces potential variability in filler dispersion and homogeneity; future studies should consider advanced mixing or ultrasonication methods to ensure consistency. Additionally, the study did not include artificial aging protocols such as thermocycling or cyclic mechanical loading, which are essential to evaluate the long-term durability of the adhesive under intraoral conditions. Oral conditions impose repeated thermal, chemical, and mechanical stresses that can degrade bond integrity over time [27].

Finally, although hydroxyapatite is widely recognized for its biocompatibility, no biological assays were conducted here. Assessment of cytotoxicity, bioactivity, and antibacterial properties will be essential for clinical translation, especially given the increasing emphasis on biomimetic and bioactive materials in orthodontics [28].

## Conclusion

Incorporation of 2 wt% n-HAp into orthodontic adhesive significantly improved SBS without altering the failure mode. The predominant enamel–adhesive failure observed is clinically favorable, as it minimizes adhesive residue on tooth surfaces and reduces the risk of enamel damage during debonding. However, further studies incorporating aging protocols and enamel hardness assessments are necessary to confirm the long-term performance and clinical applicability of this modified adhesive.

## Data Availability

The data supporting the findings of this study, specifically regarding shear bond strength and adhesive remnant score, are available from the corresponding author upon reasonable request. Due to confidentiality agreements and institutional policies, the raw datasets are not publicly accessible but can be provided to qualified researchers for verification and further research purposes.

## References

[1] Gonçalves A, Barros G, Coelho M, Monteiro F, Silva FS, Pinho T. Effectiveness of surgical and non-surgical techniques for accelerating orthodontic tooth movement in fixed appliances and aligners: a systematic review. Turk J Orthod. 2025;38(1):64–79. 10.4274/TurkJOrthod.2025.2024.102 40150886 PMC11976350

[2] Urala AS, Sathyamoorthy H, Pentapati KC. Incidence of orthodontic bracket detachment and associated factors among individuals undergoing orthodontic treatment. J Datta Meghe Inst Med Sci Univ. 2023;18(2):206–9. 10.4103/jdmimsu.jdmimsu

[3] Almosa N, Zafar H. Incidence of orthodontic brackets detachment during orthodontic treatment: a systematic review. Pak J Med Sci. 2018;34(3):744–50. 10.12669/pjms.343.15012 30034451 PMC6041531

[4] Labunet A, Kui A, Voina-Tonea A, Vigu A, Sava S. Orthodontic attachment adhesion to ceramic surfaces. Clin Cosmet Investig Dent. 2021;13:83–95. 10.2147/CCIDE.S302770 PMC7982441 33762853

[5] Scribante A, Contreras-Bulnes R, Montasser MA, Vallittu PK. Orthodontics: bracket materials, adhesives systems, and their bond strength. Biomed Res Int. 2016;2016:10–12. 10.1155/2016/1329814 PMC5081464 27818996

[6] Reynolds IR. A review of direct orthodontic bonding. Br J Orthod. 1975;2(3):171–8. 10.1080/0301228x.1975.11743666

[7] Okeke AC, Utomi I, Folaranmi N. Comparative study of the shear bond strengths and bracket failure rates of two orthodontic adhesive systems. Pesqui Bras Odontopediatria Clin Integr. 2022;22:1–12. 10.1590/PBOCI.2022.073

[8] Shams S, Abela S, Andiappan M, Hajiheshmati A, Bister D. Shear bond strengths of 3 commonly used orthodontic adhesives. dentistry. 2020;10(7):1–6. 10.35248/2161-1122.20.10.568

[9] Santos M, Fidalgo-Pereira R, Torres O, Carvalho O, Henrique B, Özcan M, et al. The impact of inorganic fillers, organic content, and polymerization mode on the degree of conversion of monomers in resin-matrix cements for restorative dentistry: a scoping review. Clin Oral Investig. 2024;28(8):1–19. 10.1007/s00784-024-05829-6 PMC11283416 39066793

[10] Hasan LA. Evaluation the properties of orthodontic adhesive incorporated with nano-hydroxyapatite particles. Saudi Dent J. 2021;33(8):1190–6. 10.1016/j.sdentj.2021.01.001 34938065 PMC8665179

[11] Hasan LA. Evaluation the properties of orthodontic adhesive incorporated with nano-hydroxyapatite particles. Saudi Dent J.2021;33(8):1190-1196. 10.1016/j.sdentj.2021.01.001 34938065 PMC8665179

[12] Mathirat A, Dalavi PA, Prabhu A, Yashaswini Y, Anil S, Senthilkumar K, et al. Remineralizing potential of natural nano-hydroxyapatite obtained from epinephelus chlorostigma inartificially induced early enamel lesion: an in vitro study. Nanomaterials.2022;12(22):1-15. 10.3390/nano12223993 PMC9693638 36432279

[13] Balhuc S, Campian R, Labunet A, Negucioiu M, Buduru S, Kui A. Dental applications of systems based on hydroxyapatite. J Crystals. 2021;11:1–19.

[14] Hikmah N, Tanumihardja M, Nugroho JJ, Natsir N, Hamrun N, Kasim S. Potential of nano hydroxyapatite synthesized from blood clam shells as a remineralizing agent after in-office bleaching. J Dentomaxillofac Sci. 2023;8(2):122–6. 10.15562/jdmfs.v8i2.1569

[15] Amalina R, Monica D, Feranisa A, Syafaat FY, Sari M, Yusuf Y. Development of hydroxyapatite Asian Moon Scallop (Amusium Pleuronectes) gel and its effect after application on tooth enamel white-spot lesion. Cakradonya Dent J. 2021;13(2):81–7.

[16] Hardianto E, Satriyo TB. Molecular phylogenetic analysis of commercially important Asian monsoon scallop, Amusium pleuronectes (Linnaeus 1758) from Indonesia. J Kelaut Tropis. 2023;26(3):442–50. 10.14710/jkt.v26i3.18049

[17] Muntean FL, Olariu I, Marian D, Olariu T, Petrescu E, Olariu T, et al. Hydroxyapatite from Mollusk Shells: characteristics, production, and potential applications in dentistry. Dent J (Basel). 2024;12(12):1–24. 10.3390/dj12120409 PMC11674191 39727466

[18] Syafaat FY, Yusuf Y. Influence of ca/p concentration on hydroxyapatite (Hap) from asian moon scallop shell (amusium pleuronectes). Int J Nanoelectronics Materials. 2019;12(3):357–62.

[19] Pamungkas GB, Karunia D, Suparwitri S. Desensitizing agents’ post-bleaching effect on orthodontic bracket bond strength. Dent J (Majalah Kedokteran Gigi). 2024;45(158):45–9. 10.20473/j.djmkg.v57.i1.p45

[20] Goracci C, Di Bello G, Franchi L, Louca C, Juloski J, Juloski J, Vichi V.Bracket bonding to all-ceramic materials with universal adhesives. Materials. 2022;15(3):1-11. 10.3390/ma15031245 PMC8839010 35161189

[21] Valluri BP, Kanumuri MV, Sajjan G, Rajulapati KS, Penmatsa VKV, Mavidi JB. Effect of nano-hydroxyapatite incorporation on the immediate and long-term bond stability of a one-step self-etch adhesive. J Dent Mater Tech. 2025;14(2):57–63. 10.22038/jdmt.2025.85728.1781

[22] Khan G, Ahmed J, Khan AS, Riaz A, Iqbal A, Hussain S. Development and evaluation of nano-hydroxyapatite and silica-reinforced dental composites: enhancing mechanical strength and hydrolytic stability. J Popul Ther Clin Pharmacol. 2025;32(02):8–15. 10.53555/nh0mwk44

[23] Yu F, Dong Y, Yu H, Lin P, Zhang L, Sun X, et al. Antibacterial activity and bonding ability of an orthodontic adhesive containing the antibacterial monomer 2-methacryloxylethyl hexadecyl methyl ammonium bromide. Sci Rep.2017;7(June 2016):1–9. 10.1038/sr 28169312 PMC5294631

[24] Kumar U, Kumar D, Gosai KN, Dalal D, Pragnya B, Nagarajan S. Effectiveness of nanoparticles in enhancing bond strength in adhesive dentistry article. J Pharm Bioallied Sci. 2024;7(10):1–5. 10.4103/jpbs.JPBS PMC11805008 39926728

[25] Loumprinis N, Maier E, Belli R, Petschelt A, Eliades G, Lohbauer U. Viscosity and stickiness of dental resin composites at elevated temperatures. Dent Mater. 2021;37(3):413–22. 10.1016/j.dental.2020.11.024 33353736

[26] Noworyta M, Topa-Skwarczyńska M, Jamróz P, Oksiuta D, Tyszka-Czizhara M, Trembecka-Wójciga M, et al. Influence of the type of nanofillers on the properties of composites used in dentistry and 3D printing. Int J Mol Sci. 2023;24(13):1-23. 10.3390/ijms241310549 PMC10341704 37445729

[27] Prasad M, Mohamed S, Nayak K, Shetty S, Talapaneni A. Effect of moisture, saliva, and blood contamination on the shear bond strength of brackets bonded with a conventional bonding system and self-etched bonding system. J Nat Sci Biol Med. 2014;5(1):123–9. 10.4103/0976-9668.127305 24678210 PMC3961916

[28] Scribante A, Dermenaki Farahani MR, Marino G, Matera C, Rodriguez y Baena R, Lanteri V, et al. Biomimetic effect of nano-hydroxyapatite in demineralized enamel before orthodontic bonding of brackets and attachments: visual, adhesion strength and hardness in in vitro tests. Biomed Res Int. 2020;2020:1-9. 10.1155/2020/6747498 PMC7013302 32090106

